# Ultra-wide field swept-source optical coherence tomography angiography in patients with diabetes without clinically detectable retinopathy

**DOI:** 10.1186/s12886-021-01933-3

**Published:** 2021-05-01

**Authors:** Jingyuan Yang, Bilei Zhang, Erqian Wang, Song Xia, Youxin Chen

**Affiliations:** 1 Department of Ophthalmology, Peking Union Medical College Hospital, Chinese Academy of Medical Sciences, No.1 Shuaifuyuan Wangfujing, Dongcheng District, Beijing, 100730 China; 2 Key Laboratory of Ocular Fundus Diseases, Chinese Academy of Medical Sciences, No.1 Shuaifuyuan, Wangfujing, Dongcheng District, Beijing, China; 3 Department of Ophthalmology, Guizhou Provincial People’s Hospital, Guiyang, China

**Keywords:** Diabetic retinopathy, Nonperfusion area, Optical coherence tomography angiography, Neovascularization

## Abstract

**Background:**

To investigate alterations in retinal microvasculature in eyes with preclinical diabetic retinopathy (DR) using ultra-wide field swept-source optical coherence tomography angiography (UWF SS OCTA).

**Methods:**

Prospective cross-sectional study. Fifty-five eyes of 30 diabetic patients without clinical retinal signs were included. All subjects underwent OCTA examination with a 12 × 12 mm^2^ field of view of 5 visual fixations (1 central fixation and 4 peripheral fixations) to compose a UWF OCTA image. In the UWF images, the central area corresponded to the original central image obtained using central fixation, and the peripheral area was the remaining area. Lesions, including nonperfusion areas (NPAs), microvascular dilation and tortuosity, and neovascularization (NV), were recorded in different areas. Diabetes history was also recorded.

**Results:**

Peripheral areas presented significantly more microvascular dilation and tortuosity than central areas (*P* = 0.024) and more NPAs than central areas, with borderline significance (*P* = 0.085). The number of lesion types was associated with HbA1c levels in the peripheral and overall areas (all *P* values < 0.001).

**Conclusions:**

UWF SS OCTA is a promising imaging method for detecting vascular alterations in diabetic eyes without clinical signs to reveal retinal microvascular alterations. These alterations were correlated with systemic conditions.

**Supplementary Information:**

The online version contains supplementary material available at 10.1186/s12886-021-01933-3.

## Background

Diabetic retinopathy (DR) is the most common complication of diabetic microvascular disease, and it is the leading cause of vision impairment in the working-age population worldwide [1, 2]. These retinal microvascular changes allow clinicians to monitor diabetic complications in a more sensitive and individualized manner with shorter intervals [3–5]. Preclincal DR was defined as no clinical signs of diabetic retinopathy in diabetic patients, such as no visible microvascular or other DR-related findings upon dilated ophthalmoscopy [4, 6–10]. By taking advantage of the transparency of ocular structures and examining the retina in vivo, early detection of retinal microvascular changes in preclinical DR can be used to recognize microvascular changes due to long-term exposure to hyperglycaemia and to manage patients at a greater risk of DR progression [11]. The use of functional and structural retinal changes as novel preclinical biomarkers allows the recognition and investigation of diabetic microvascular disease and retinopathy.

A non-invasive examination, optical coherence tomography angiography (OCTA), is currently the predominant method for detecting microvascular changes in the retina; it has challenged the use of fluorescein angiography (FA) in some clinical practices because of the prevention of invasion and severe life-threatening reactions, including anaphylaxis, cardiac arrest, and bronchospasm [12]. Several studies have reported that microvascular changes, such as retinal capillary dropout, foveal avascular zone irregularity, and capillary tortuosity, were noticed in approximately 31 to 58% of eyes with preclinical DR. [7, 9] However, all of these studies used scan patterns with limited scanning areas no larger than 6 × 6 mm^2^, and potential peripheral lesions were not well detected [13, 14]. .Currently, a larger field of view (FOV) of up to 12 × 12 mm^2^ can be achieved in a single scan by utilizing a commercial swept-source (SS) OCTA system. Several studies have confirmed the feasibility of using an SS OCTA system to visualize alterations on ultra-wide field (UWF) montage images of DR. [15–18] Therefore, UWF OCTA images could also be used to detect microvascular changes in eyes with preclinical DR and have potential utility for screening for DR.

The purpose of the present study was to investigate changes in retinal microvasculature in eyes with preclinical DR using UWF SS OCTA and to investigate the relationship between retinal microvascular alterations and systemic conditions.

## Methods

### Subjects

This prospective cross-sectional study was performed in accordance with the tenets of the Declaration of Helsinki. This study was approved by the Institutional Review Board of Peking Union Medical College Hospital (approval number ZS-1976), and informed consent was obtained from all the participants.

Patients with type 2 diabetes mellitus (T2DM) were recruited prospectively. The diagnosis of T2DM was made by an endocrinologist according to the diagnostic criteria of the American Diabetes Association [19]. For the diabetic eyes, the diagnosis of DR and classification of retinal microvascular lesions in OCTA images were made by two retinal specialists (JY and BZ). In cases of disagreement, a third retinal specialist (YC) made the final decision. The inclusion criteria for diabetic patients were as follows: normal anterior segment, and the absence of microaneurysms, haemorrhages, or ophthalmoscopically detectable evidence of capillary nonperfusion or retinal neovascularization. The exclusion criteria were as follows: (1) eyes with other ocular diseases or a previous history of surgeries that affect retinal vessels; (2) history of systemic disease other than DM that affect the eyes; (3) intraocular pressure > 21 mmHg; and (4) poor-quality OCTA images.

### Examinations

The subjects underwent a comprehensive ocular examination that included best-corrected visual acuity, intraocular pressure, slit lamp fundus examination with a non-contact lens, fundus examination with direct ophthalmoscopy, optical coherence tomography (OCT) and OCTA, and UWF imaging using the Optos 200Tx system (Optos PLC, Dunfermline, Scotland). The UWF images were used to confirm the absence of clinical signs because it is frequently used for UWF colour imaging of nearly the entire retina (up to 200 degrees) at one time without changing the fixation target. The UWF imaging procedure included at least one 200° image centered on macula.

Three-dimensional OCTA scans of 12 × 12 mm^2^ regions were acquired using a commercial SS OCTA system (VG200, SVision Imaging, Ltd., Luoyang, China). The commercial SS OCT device contained an SS laser with a central wavelength of approximately 1050 nm (990–1100 nm full width) and a scanning rate of 20 k A-scans per second. The device had a full-width at half maximum axial resolution of approximately 5 μm in tissue and an estimated lateral resolution at the retinal surface of approximately 15 μm. Volume data, including both OCT and OCTA data, were obtained with a raster scan protocol of 1024 (horizontal) × 1024 (vertical) B-scans repeated twice at each of 1024 B-scan positions, which takes approximately 12 s per scan with an artificial-intelligence-assist tracking system. For subjects who were unable to maintain the position of fixation and whose images had excessive motion artifacts, a raster scan protocol of 768 (horizontal) × 768 (vertical) B-scans was used, which takes approximately 7 s per scan (Supplementary Figure 1). OCTA scans were obtained at five fixation points (central, inferior nasal, superior nasal, inferior temporal, and superior temporal) to obtain a wider field of view (see Supplementary Figure 2 for a DR eye and Supplementary Figure 3 for a normal eye). A composite image could then be generated from five OCTA images and superimposed on UWF colour images using built-in software (Supplementary Figure 2) [16]. The composite UWF OCTA image was then divided into 2 areas: the central area corresponded to the central OCTA image of the 5 small images, and the rest of the composite UWF OCTA image was defined as the peripheral area. By using this partition method to compare the central area and peripheral area, we could easily compare the traditional 12 × 12 mm2 OCTA image with the peripheral area, which provides additional information that a UWF OCTA image can provide but a single individual central OCTA cannot.

Retinal microvascular lesions were categorized into 3 types: retinal nonperfusion areas (NPAs), retinal capillary dilation and tortuosity, and neovascularization (NV) [20]. NPA, which represents retinal capillary occlusion or dropout, could be identified as a dark area with discontinuous capillary or decreased capillary density on OCTA images. Retinal capillary dilation and tortuosity, including dilated capillaries with aneurysmal ends, intraretinal microvascular abnormalities (IRMAs), and venous beading, could be identified as tortuous vessels, which are usually located adjacent to nonperfusion areas within the neuroretina and are more dilated than adjacent capillaries. NV can be identified by abnormal new vessels that protruded towards the vitreous cavity, and OCTA B-scan images can assist in confirming the location of the abnormal vessels. The retinal layers were segmented using the default setting, and lesions above the choroid were counted.

### Statistical analysis

Continuous variables are presented as the mean (standard deviation, SD). Categorical variables are given as numbers and percentages. Pairwise comparisons were investigated with paired *t*-tests. Multiple logistic regression analysis was performed for clinical variables (duration of diabetes, HbA1c level, diabetes treatment regime as independent variables) adjusted for age at baseline and sex [21], and the number of detected lesion types, including retinal NPAs, capillary dilation and tortuosity, and NV, was used as the dependent variable. Only right eyes were used for the multiple logistic regression analysis. To improve the the explanation and power of the analysis, the variables were grouped according to the results of previous clinical studies. Considering the demographics of the current study and the risk factors for DR occurrence in previous studies, the duration of diabetes was classified into 2 groups, < 7 years and ≥ 7 years [22, 23]; HbA1c level was classified into 2 groups, < 7%and ≥ 7% [24, 25]; and treatment was classified into 2 groups, no treatment and treatment. Statistical analyses were performed using SPSS software version 25.0 (IBM-SPSS, Chicago, IL, USA). Results with *P* < 0.05 were considered statistically significant.

## Results

Fifty-five eyes of 30 diabetic patients without clinical retinal signs were imaged for the study. The participants’ characteristics are summarized in Table 1.

**Table 1:** Clinical characteristics of participants

Characteristics	
Number of patients, n	30
Age, years (SD)	55.49 (12.55)
Sex, female/male	14/16
Eye, right/left	28/27
Duration of diabetes, years (SD)	13.73 (8.07)
HbA1c, % (SD)	8.63 (2.00)
Diabetes treatment	
No drug therapy, n (%)	4 (13.3%)
Oral hypoglycaemic agents, n (%)	5 (16.7%)
Insulin, n (%)	6 (20%)
Insulin and oral hypoglycaemic agents, n (%)	15 (50%)
Best-corrected visual acuity, logMAR (SD)	0.14 (0.21)

*HbA1c* haemoglobin A1c, *logMAR* logarithm of the minimal angle of resolution, *SD* standard deviation

**Fig. 1:**
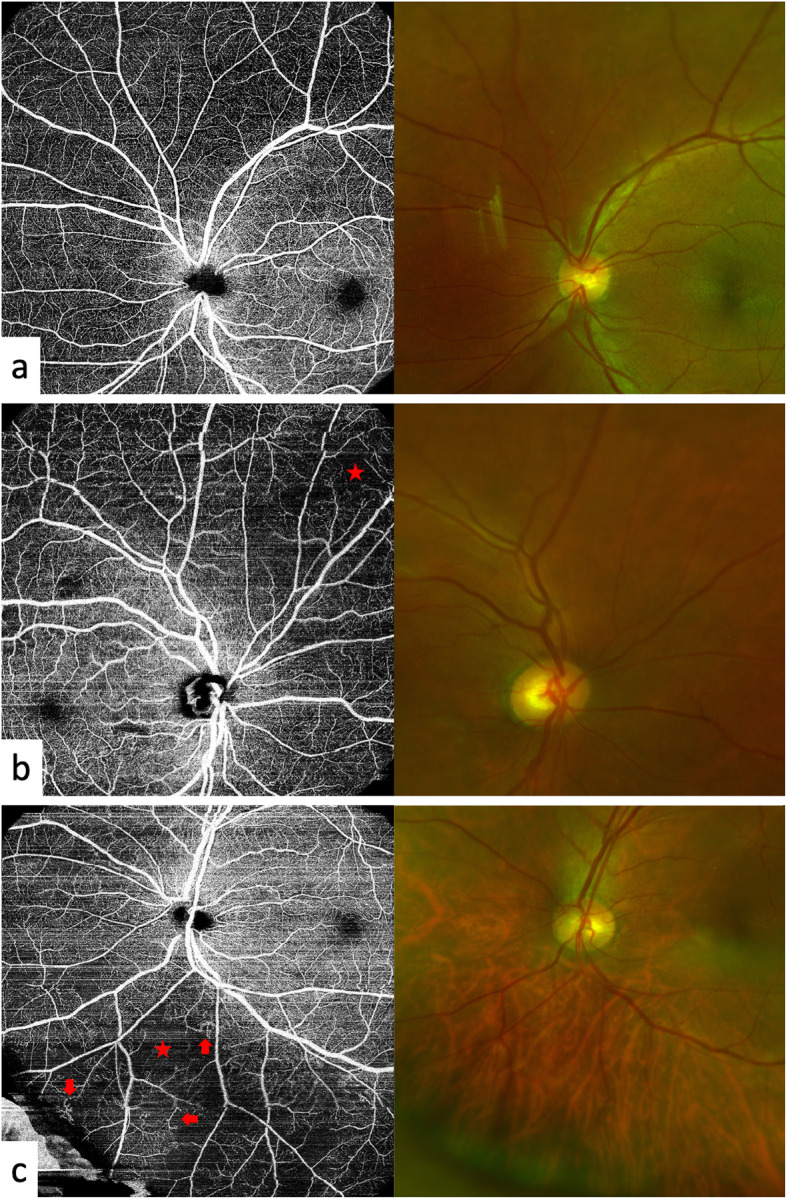
Representative peripheral optical coherence tomography angiography (OCTA) images. Only OCTA images of the superficial retinal capillary plexus are shown here to better visualize the lesions. These cases showed no significant clinical signs of diabetic retinopathy on colour fundus photos. **a** OCTA of a normal fundus of a diabetic eye without clinical signs. **b** A nonperfusion area (NPA) (asterisk) is shown in the superior nasal quadrant. **c** Both a NPA (asterisk) and capillary tortuosity (arrows) could be detected in the inferior nasal quadrant

**Fig. 2:**
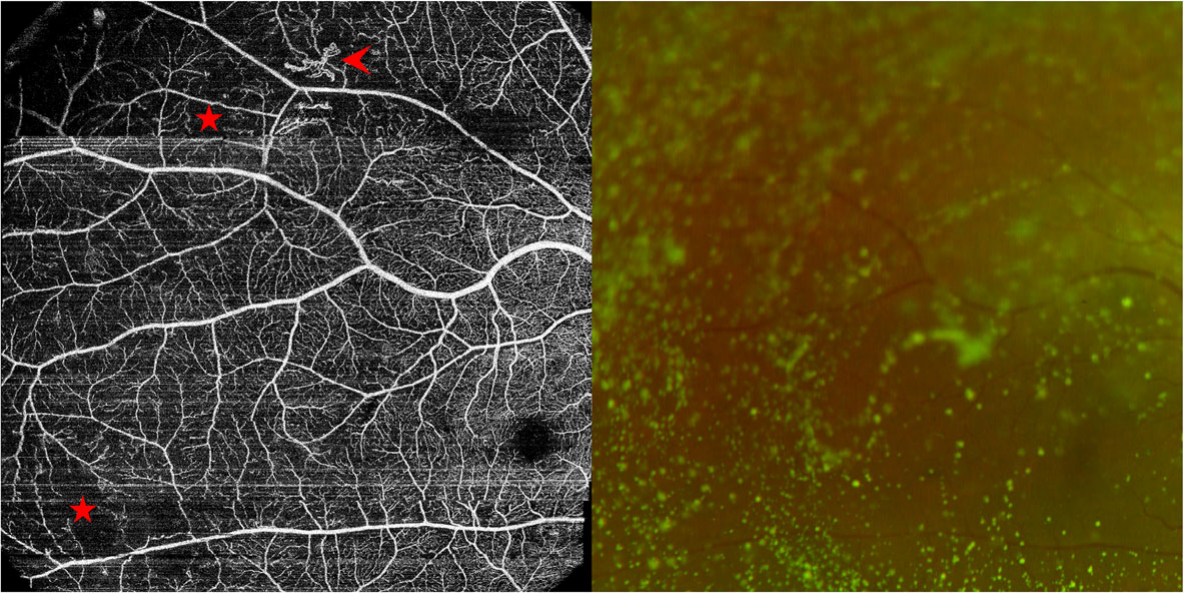
Representative cases showing the superiority of optical coherence tomography angiography (OCTA) for detecting vascular lesions that are difficult to detect with conventional non-invasive fundus examinations. A 63-year-old female had vitreous asteroid hyalosis, which made retinal abnormalities difficult to detect with non-contact lens and direct ophthalmoscopy. Swept-source OCTA images showed not only NPAs (asterisks) but also neovascularization (arrowhead)

Although no clinical signs were detected in the enrolled eyes by non-contact lens and direct ophthalmoscopy, UWF OCTA revealed retinal microvascular lesions in 32 eyes (58.2%) (detailes are shown in Fig. 1, representative images are shown in Supplementary Figure 4). NPAs were more frequently detected in the peripheral areas than in the central areas, and the difference was borderline significance (*P* = 0.085) (Table 2). Capillary dilation and tortuosity were significantly more frequently detected in the peripheral areas than in the central areas (*P* = 0.024) (Table 2). An NV lesion was detected in one eye that was excluded because of vitreous asteroid hyalosis that made it difficult to detect retinal abnormalities before OCTA examinations (Fig. 2).

**Table 2:** Locations of lesions detected in various retinal areas by UWF OCTA

Lesions	Lesions in central areas	Lesions in peripheral areas	*P* value
Nonperfusion area, eyes (%)	21 (38.2%)	30 (54.5%)	0.085
Capillary dilation and tortuosity, eyes (%)	3 (5.5%)	12 (21.8%)	0.024
Neovascularization, eyes (%)	0 (0)	0 (0)	–

*UWF OCTA* ultra-wide field optical coherence tomography angiography

Because 3 types of microvascular lesions were identified, the number of lesions was between 0 and 3. In the multiple logistic regression analysis results after controlling for age and sex, neither the duration of diabetes, the HbA1c level, nor the treatment regimen was significantly associated with the number of lesion types in the central areas (all *P* values > 0.05). Only the HbA1c level showed a significant association with the number of lesion types in peripheral areas (*P* = 0.004, OR was 1.880 × 10^9^ for the presence of 1 type of lesion and 4.143 × 10^8^ for the presence of 2 types of lesions) and overall (*P* = 0.008, OR was 1.449 × 10^9^ for the presence of 1 type of lesion and 5.731 × 10^8^ for the presence of 2 types of lesions).

## Discussion

In the current study, an SS OCTA system was used with a novel protocol to render UWF OCTA images. We found that microvascular impairments, including capillary dropout, NPAs, IRMAs, and NV, had already started in the eyes without clinical signs. These impairments were detected more often in peripheral regions than in macular regions. These microvascular lesions were associated with HbA1c levels.

SS OCTA devices usually have better penetration and faster scanning speeds than spectral-domain OCT devices, which leads to a better image quality for eyes with cloudy refracting media and a shorter scan time. With a determined scanning area, a corresponding increase in the resolution for imaging the fine retinal vasculature was achieved with faster scanning speed. A larger scanning range of depth was needed for the peripheral retina and choroid than for the macular retina and choroid, and the roll-off performance of SS OCTA enables visualization of the peripheral retina and choroid. The faster speed of the current SS OCTA system (20 kHz) and its artificial-intelligence-assisted tracking system enable extension of the FOV with reduced scanning time. Therefore, SS OCTA devices can currently be applied to detect microvascular changes in eyes without clinical signs of DR on UWF OCTA images.

In the current study, UWF SS OCTA showed good performance for detecting retinal microvascular impairment in diabetic eyes without clinically detectible signs, which suggests that this procedure may be useful for detecting vascular lesions in diabetic patients. Routine fluorescein angiography is not indicated as a part of the regular examination of diabetic patients, especially those without clinical retinal signs [26]. A more sensitive screening method for retinal vascular alterations is needed. Several studies have reported the potential use of OCTA as a clinical tool for earlier objective detection of preclinical DR. [4, 6–10] An enlarged and irregular foveal avascular zone, capillary dropout, microaneurysm, capillary dilation, NPAs, and decreased vessel density were captured by OCTA in diabetic eyes without clinical signs. Cao et al. reported that NPAs could be found in 25.4% (18/71) of preclinical DR eyes of patients with T2DM using a scanning protocol of 6 × 6 mm^2^ with a spectral-domain OCTA device [6]. In the present study, NPAs were found in 39.3% of eyes using a scanning protocol of 12 × 12 mm^2^ with the SS OCTA device, and they were found in 55.4% of the eyes when the FOV was widened with peripheral OCTA images. In the present study, other lesions, including capillary dilation and tortuosity and NV, were also more commonly noticed in peripheral OCTA images, which indicates that a larger OCTA scanning area could provide more evidence of retinal microvascular lesions in the early stage of DR. However, the influence of the lesions on DR onset and progression is unknown. Therefore, longitudinal studies are needed to investigate the feasibility of using UWF SS OCTA as a regular screening tool.

In the current study, retinal microvascular lesions were classified into 3 types: NPAs, capillary dilation and tortuosity, and NV. The development of capillary segment nonperfusion in diabetic eyes before clinical DR is evident [4, 27]. Hypoxia is thought to be an early inciting event in the pathogenesis of retinal microvascular lesions in DR eyes. It could result in an increase in vessel calibre and a decrease in elasticity of the vessel wall [28–30]. Adjacent to areas with apparently retinal ischaemia, microvascular remodelling, such as IRMAs, occurs. Retinal ischaemia also plays a central role in the pathogenesis of NV by stimulating the elaboration of vascular endothelial growth factor and other angiogenic factors [31, 32]. Therefore, the classification of lesion types in the present study was in accordance with the pathogenesis of DR, and the number of lesion types reflected the severity of diabetes to some extent. However, quantitative metrics, such as vessel density and nonperfusion area, would be more precise than the metrics used in the current study.

In the present study, we investigated the relationship between systemic conditions and the number of lesion types detected by OCTA. We used the number of lesion types rather than the lesion area as the metric in the present study. Because of azimuthal projection, UWF images cannot be simply mapped onto a flat surface, such as a two-dimensional OCTA image, without warping [33]. To the best of our knowledge, there is no accurate way for commercial OCTA devices to determine the precise area of lesions on UWF SS OCTA images. Instead, we analysed the severity of retinal microvascular lesions semi-quantitatively by calculating the number of lesion types. The simultaneous use of both central and peripheral images to evaluate retinal microvasculature should be more accurate and comprehensive than the use of central images alone. The regression analysis performed in the current study suggests that the peripheral OCTA images and the whole OCTA images showed a similar association with HbA1c level, and their β values in the regression analysis were greater than the β value when only central OCTA images were used. However, the correlation of changes in wide-field OCTA images and system conditions needs further validated in prospective studies of greater sample size.

In the current study, the concept of ‘preclinical DR’ was confirmed by fundus examinations through dilated pupils and colour photos. Our results, together with those of Tam and Burns [34, 35], make a strong case that the current definition and classification of DR based on lower-contrast imaging methods provide insufficient specific information about the retinal capillaries. UWF SS OCTA makes it possible to distinguish among patients who are undergoing retinal microvascular changes that might threaten visual acuity. For example, in the eye with vitreous asteroid hyalosis (Fig. 2), no significant lesions were detected by ophthalmologists or with colour fundus photos. However, NV was detected by OCTA, which suggested that OCTA was superior to colour fundus photos for detecting microvascular alterations. Because of its advantages of high resolution, convenience, and non-invasiveness, UWF OCTA might be helpful for detecting microvascular changes at an early stage. However, the role of UWF OCTA in the diagnosis and management of DR needs to be further investigated.

The influence of peripheral lesions observed with UWF OCTA on visual acuity, which is primarily determined by the central retina, needs further investigation, although the peripheral lesions observed in the current study seem less likely than central lesions to threaten visual acuity, at least not in the short term. However, peripheral lesions observed with UWF images, which covers a larger area than the UWF OCTA images in the present study, have a correlation with DR progression over the long term [36]. Some severe peripheral lesions observed with OCTA, such as IRMAs and NV, might need to be further evaluated and confirmed using fluorescein angiography [7]. Therefore, the findings with UWF OCTA may help to characterize patients in the preclinical stage of DR. Longitudinal studies would be helpful for investigating the influence of these findings on the management and visual prognosis of diabetic patients.

The limitations of this study most notably include the sample size and the lack of universal consensus or guidelines for UWF OCTA. Given the small sample size, it was difficult to draw firm conclusions from the regression analysis. Studies with larger sample sizes are needed to confirm the results in the future. In addition, although UWF OCTA could be used for DR, as previous studies have reported [15, 16], the pre-process of UWF OCTA images before interpretation, including image montages and retinal layer segmentation, has not been fully studied and standardized by international consensus or guidelines. UWF OCTA images should also be compared with standard 7-field fundus photographs. Instead, we used UWF images to match UWF OCTA images, which had acceptable agreement with fundus photographs of diabetic eyes without clinical retinal signs [37–39]. However, the alterations in OCTA images might be features in the healthy population due to the lack of control group without diabetes. At the same time, because the included patients had relatively normal fundi, FA was not performed. As a result, the nature of some lesions, including microaneurysms and intraretinal microvascular abnormalities, could not be confirmed accurately. Additionally, more information about the demographics of the study population, including blood pressure, cardiovascular health, and more laboratory data, might help evaluate these elements more comprehensively in future studies to elucidate the associations between UWF OCTA images and systemic conditions. More importantly, quantitative metrics for interpreting UWF OCTA images should be introduced by OCTA device manufacturers to enable the more precise evaluation of the retinal microvasculature. The quality of the figures in the current study could be better. With the development of the OCTA technique, the image quality will surely improve significantly, and more retinal and choroidal alterations might be imaged better.

## Conclusions

OCTA is a promising non-invasive and high-resolution imaging method that has the potential to reveal retinal microvascular alterations in diabetic eyes without clinical signs. The occurrence of retinal microvascular alterations was associated with HbA1c levels. Retinal microvascular lesions occurred more often in the region of the peripheral retina, a region that should be assessed carefully in diabetic patients with high HbA1c levels. This cross-sectional study is a first step towards the understanding of subtle vascular changes in diabetic eyes without clinical signs. The relationship between prognosis and preclinical microvascular alterations needs to be investigated in a longitudinal study.

## Supplementary Information

**Additional file 1: Supplementary Figure 1.** Examples of 12 × 12 mm^2^ optical coherence tomography angiography images of resolution of 1024 × 1024 (**a**) and 768 × 768 (**b**). Both of these images shows fine retinal capillary network.

**Additional file 2: Supplementary Figure 2.** Ultra-wide field optical coherence tomography angiography (UWF OCTA) images of an eye with moderate non-proliferative diabetic retinopathy after pupillary dilation. One central and 4 peripheral images (**a**) were combined to generate a composite image (**b**), whose field of view (FOV) is enclosed by a dashed-line square in the UWF colour fundus photograph (**c**). The FOV of each single image (**a**) was 12 × 12 mm^2^. The central image included the optic disc and its major retinal arteries and veins, and the peripheral images widened the detected FOV. The peripheral vessel density was significantly lower than the central vessel density in the eye with diabetic retinopathy.

**Additional file 3: Supplementary Figure 3.** Composite ultra-wide field optical coherence tomography angiography (UWF OCTA) image of a normal eye of a 25-year-old female without pupillary dilation. No significant decrease in vessel density was detected. Peripheral artefacts were generated by the undilated pupil.

**Additional file 4: Supplementary Figure 4**. Ultra-wide field optical coherence tomography angiography (UWF OCTA) images of preclinical diabetic retinopathy after pupillary dilation. NPAs (asterisk) and capillary tortuosities (arrows) could be detected in UWF OCTA images.

## Data Availability

The datasets used and/or analysed during the current study are available from the corresponding author on reasonable request.

## Abbreviations

DR: Diabetic retinopathy
FOV: Field of view
FA: Fluorescein angiography
IRMA: Intraretinal microvascular abnormality
NV: Neovascularization
NPA: Non-perfusion area
OCT: Optical coherence tomography
OCTA: Optical coherence tomography angiography
SD: Standard deviation
SS: Swept-source
T2DM: Type 2 diabetes mellitus
UWF: Ultra-wide field
