# Supplementary figures and images for: Ultra-wide field swept-source optical coherence tomography angiography in patients with diabetes without clinically detectable retinopathy
Source: BMC Ophthalmol. 2021 May 1;21:192. doi: 10.1186/s12886-021-01933-3 (PMC8088031; doi:10.1186/s12886-021-01933-3)

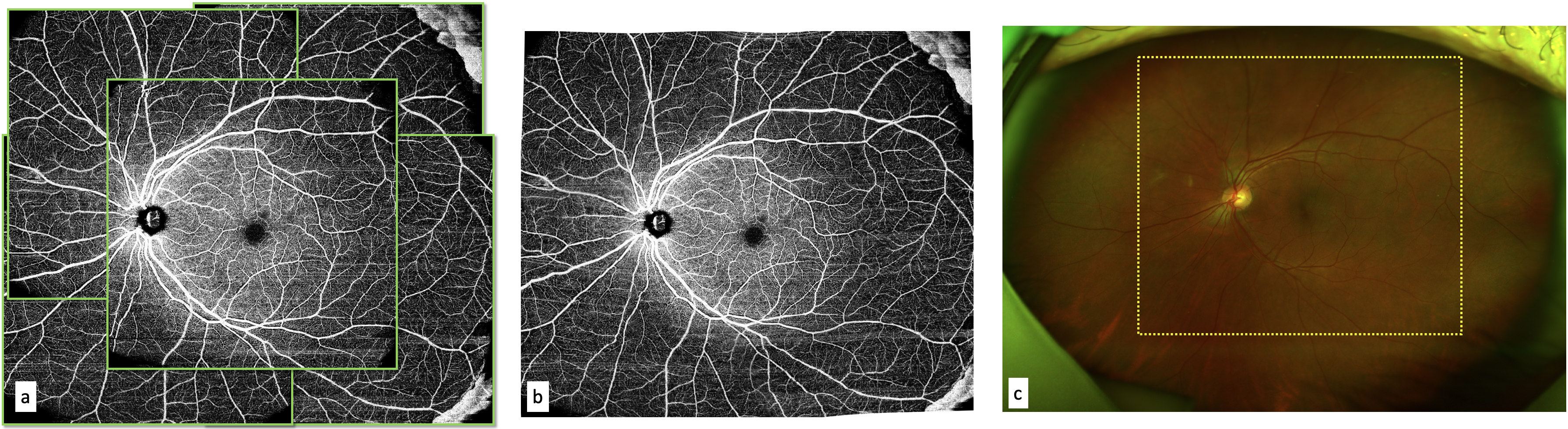

Supplement: Supplementary file 2 — Additional file 2: Supplementary Figure 2. Ultra-wide field optical coherence tomography angiography (UWF OCTA) images of an eye with moderate non-proliferative diabetic retinopathy after pupillary dilation. One central and 4 peripheral images (a) were combined to generate a composite image (b), whose field of view (FOV) is enclosed by a dashed-line square in the UWF colour fundus photograph (c). The FOV of each single image (a) was 12 × 12 mm2. The central image included the optic disc and its major retinal arteries and veins, and the peripheral images widened the detected FOV. The peripheral vessel density was significantly lower than the central vessel density in the eye with diabetic retinopathy. [file 12886_2021_1933_MOESM2_ESM.tif]

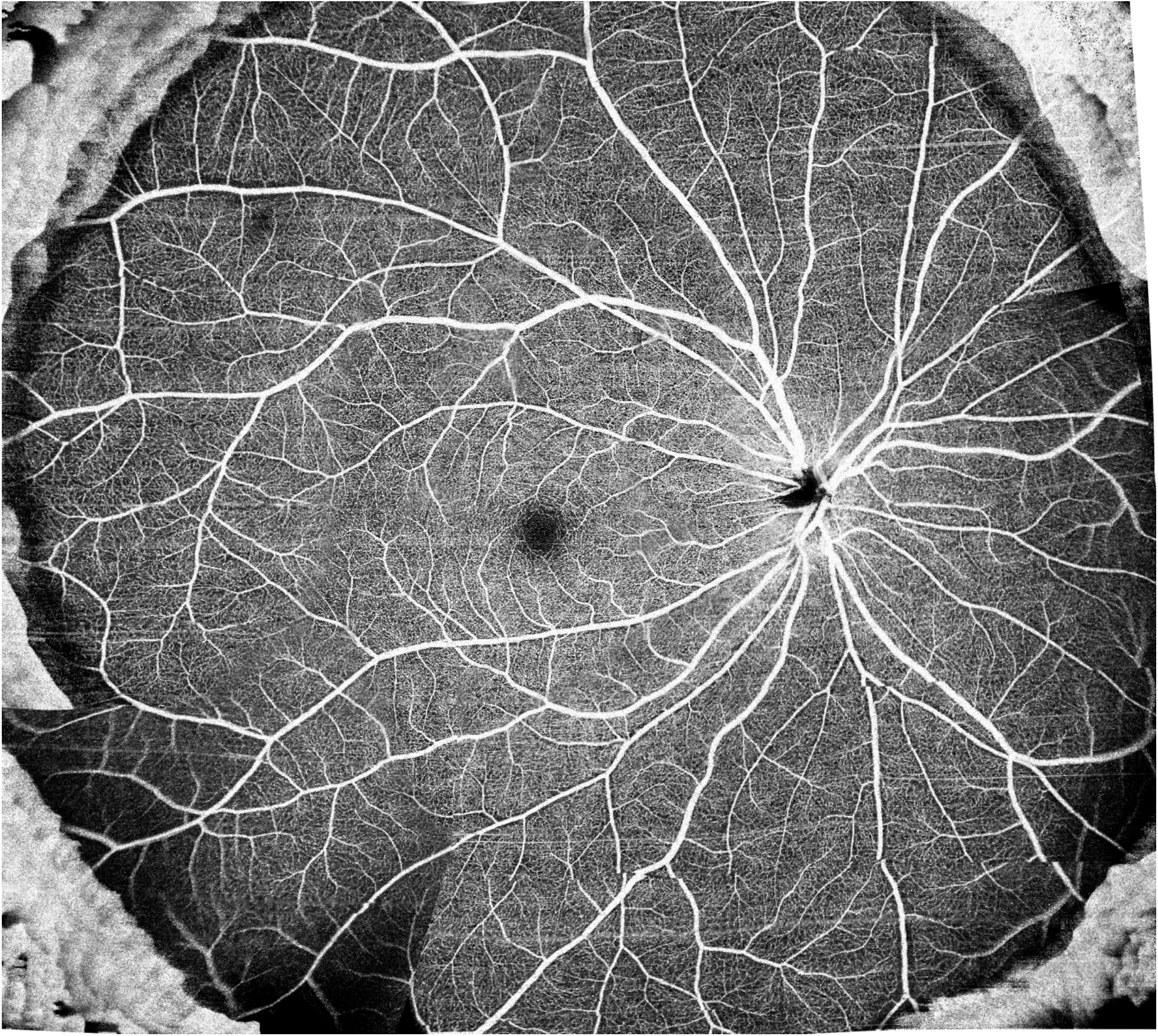

Supplement: Supplementary file 3 — Additional file 3: Supplementary Figure 3. Composite ultra-wide field optical coherence tomography angiography (UWF OCTA) image of a normal eye of a 25-year-old female without pupillary dilation. No significant decrease in vessel density was detected. Peripheral artefacts were generated by the undilated pupil. [file 12886_2021_1933_MOESM3_ESM.tif]

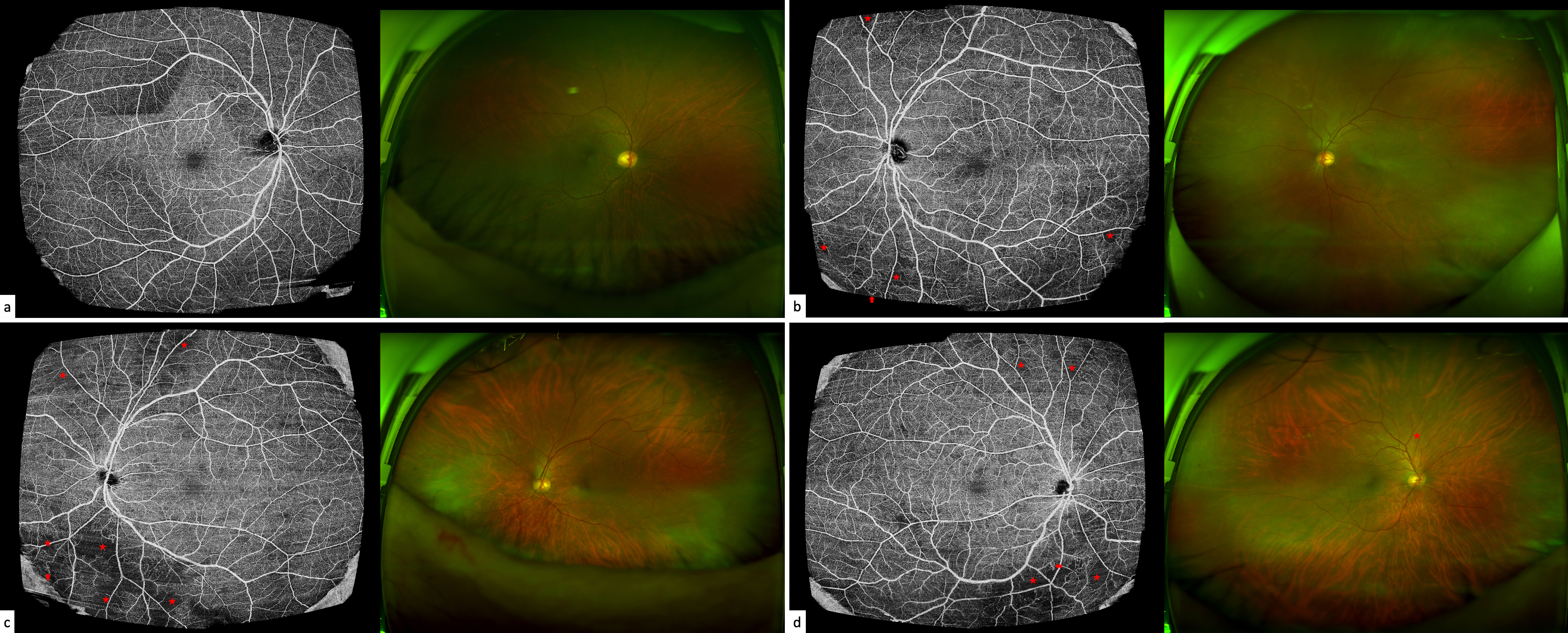

Supplement: Supplementary file 4 — Additional file 4: Supplementary Figure 4. Ultra-wide field optical coherence tomography angiography (UWF OCTA) images of preclinical diabetic retinopathy after pupillary dilation. NPAs (asterisk) and capillary tortuosities (arrows) could be detected in UWF OCTA images. [file 12886_2021_1933_MOESM4_ESM.tiff]
